# Statistics education at a time of global disruption and crises: a growing challenge for the curriculum, classroom and beyond

**DOI:** 10.1007/s41297-022-00167-7

**Published:** 2022-07-11

**Authors:** Jane Watson, Caroline Smith

**Affiliations:** 1 School of Education, University of Tasmania, Hobart, TAS Australia; 2 School of Education, University of Tasmania, Burnie, TAS Australia

**Keywords:** Statistics education, Data, COVID-19, Climate change, Statistical literacy

## Abstract

COVID-19 provided the world at large, including the world of mathematics education, with a challenge demanding attention and understanding. If met, this could potentially provide society with many of the skills needed to tackle the challenges of hyperobjects (Morton, 2013) such as climate change, which are potentially more threatening in the long-term. The COVID-19 context and the massive amount of data it has produced are the most recent examples of the growing recognition that the school mathematics curriculum has a role to play outside of the pure mathematics classroom. This paper considers COVID-19 as a stimulus for increasing the importance of statistical literacy and data literacy in preparing society for coping with world crises. Topics considered include the importance of acknowledging statistics as a significant component of mathematical ways of knowing, the contextual motivation provided by the COVID-19 crisis, the importance of statistics and statistical literacy, the place of statistics in the wider school curriculum, and finally, its place in the classroom. These topics need to be taken into account by both policy makers and teachers.

## Background stimulus

As humanity enters the second decade of the twenty-first century in the age of the Anthropocene, it becomes abundantly clear that the world has arrived at what Steger and James (2020) have described as “the Great Unsettling” — a time where the “intensifying dynamics of instability, disintegration, insecurity, dislocation, relativism, inequality, and degradation … (are) unprecedented in their compounding confluence of events” (p.188). Climate change and the COVID-19 pandemic are the most visible manifestations of this period, examples of what Morton (2013) has termed “hyperobjects” — phenomena that are massively distributed in time and space relative to the human scale and that threaten the future of humanity. They emerge from a confluence of complex events stemming from the massive disruption of the natural environment that has put unbridled unsustainable economic growth as a political imperative, driven by development of technology and powered by fossil fuels (Anderson, 2020; Bollier, 2020; Kolinjivadi, 2020; Thiele, 2013).

The question is whether such hyperobjects can teach society lessons that will assist in responding to these challenges that threaten the future of humanity. Smith and Krabbe (2019) argue that there is clearly a sense of urgency required in our response to the crisis of the Anthropocene that calls for new thinking and knowledge practices. Education at all levels has a critical role to play in conceptualising and mobilising ways to address the crises. In this paper, we ask what role can mathematics education and in particular, statistics education, play in influencing educational policy and practice to prepare a generation of citizens to change direction and make meaningful decisions in the face of continuing and future global crises? Because statistical analyses cannot exist without context (e.g., Rao, 1975), we propose that statistical literacy and data literacy can make a significant contribution to statistics education and provide recent reinforcement of Skovsmose’s (1994) critical mathematical ways of knowing. The COVID-19 crisis context has provided massive amounts of data and types of representations that have exposed society’s need for statistical understanding, and the appreciation of statistics in global contexts related to climate change and pandemics offers promise for solutions addressing these global challenges.

## Expanding ways of knowing

These times raise a question of the form of education that is being provided to students to engage meaningfully with critical issues outside of the classroom. Speaking in the broad field of mathematics, Skovsmose (1994) discussed “critical mathematics education” as expanding on three types of mathematical knowing: the traditional mathematical skills, the technological competence in model building, and the reflection required in evaluating the applications of mathematics in society. D’Ambrosio (1990) and Gutstein (2006) went further, placing mathematics education at the heart of building a democratic and equitable society. D’Ambrosio noted the threats to humankind from energy starvation, pollution, and “civilization diseases” such as AIDS, a precursor to COVID-19. Gutstein applied his belief about “reading and writing the world with mathematics” to teaching middle school mathematics in a school in a low social economic suburb of Chicago, most of whose students were of Mexican heritage. Frankenstein (2012) highlighted the “quantitative form” of arguments in a range of social contexts drawing on different formats, including cartoons, which reveal the glaring discrepancies in society. In doing so, these authors explicitly aligned mathematics education, as Frankenstein puts it, with critical analyses of social, economic, cultural, and political issues and with movements for social change.

For Pepper and Burton (2020), the COVID-19 pandemic has also been accompanied by what they term an alarming “infodemic” (an epidemic of both accurate and inaccurate information), which adds a further level of complexity to an already complex situation. Although some information is clearly true and evidence-based, other information may be completely wrong or even dangerous, confounding public trust in the scientific and medical research being carried out. One outcome is that people may clutch at simple solutions, with former US President Donald Trump’s idea of injecting disinfectant for COVID-19 being an extreme and very dangerous example (Smith, 2020). For Pepper and Burton, society’s responsibilities lie in finding accurate and plausible scientific answers and in communicating them clearly. This relies on ensuring that information is not misrepresented or misunderstood, which in turn relies on a high level of statistical literacy within the populace. In a similar vein, Cook (2019) considers in depth the misinformation being propagated about climate change and ways of countering it.

As Doherty (2015) warns, expanding ways of knowing can lead to “knowledge wars”, where commercial or political interests may lead to the questioning of scientific results based on propaganda and spin. We believe that the situation outlined presents an unprecedented and excellent opportunity and motivation for mathematics educators to engage more fully with statistics, teaching not only the techniques but also the questioning of how data are collected, what is measured, what are the contexts when comparisons are made, and who is behind an investigation. We argue that recognising the critical importance of statistics education, and statistical and data literacy across the curriculum has not only been essential for getting to grips with the complexities of issues like the COVID-19 pandemic but also in preparing and equipping the next generation for informed decision-making in the uncertain contexts they will encounter during their schooling and into the future.

## Data and COVID-19

It was not difficult to identify the presence of data and statistics in the daily news about COVID-19; in fact, it was more difficult to avoid it! At a basic level, individual statistics as data reported numbers of cases, deaths, and recoveries associated with COVID-19, as well as vaccine distribution rates and take-up rates by sections of the population. They also reported percentages of populations of countries, states, provinces, and continents. With more complexity, for example, they reported infections in terms of the average number of people an infected person would pass the virus on to, designated the R-number. An R-number less than 1.0 was “good”, but the closer to 0, the better. An R-number greater than 1.0 was “bad”, as it implies there would be increasing numbers of infected people. Issues related to the sampling techniques used, to the criteria for defining data values, and to the differing contexts where data were collected and statistics reported, however, often were not addressed.

The visualisation of actual data then took over the media spotlight with many different graphical representations employed. The choice depended on the message of the day or even the particular tactic of the presenter. The Australian Broadcasting Corporation (Ting, et al., 2020) provided an initial survey of many types of graphs used to present various relevant data collected on COVID-19 within Australia, with constant updates over time. The variety of representations went well beyond what is traditionally taught in the classroom, often pointing out the potential confusion about what was being presented, e.g. frequencies or percentages, linear or logarithmic scales, or maps with coloured circles or other area representations of outbreaks. On the international level, the best-known provider of coronavirus data has been Worldometer, which delivers a wealth of data in the form of tables, pie charts, and graphs on a daily basis (http://www.worldometers.info).

## Data and climate change

Similar to the pandemic, the field of climate science is also described and supported by a bewildering amount of data (e.g. Intergovernmental Panel on Climate Change, 2018; The World Bank, 2020). The statistics used in discussing and understanding climate change typically include atmospheric carbon dioxide and other greenhouse gas concentrations expressed in parts per million (ppm) and atmospheric temperatures rises. Other climate data describe energy and resource usage and emissions variously expressed in mass or energy units, percentages, or as graphical representations. Many of these data require clear interpretation for lay people to understand the issues, otherwise the data can be interpreted in a number of ways that feed into belief systems to support or deny the existence or severity of climate change.

Indeed, it is to be regretted that over the past decade, just as the need for understanding has become critical, scientists and mathematicians seem to have lost status in some parts of society, to the extent that experts are routinely ridiculed in some circles, and all opinion is held to have equal value (Miller, 2020). Ignorance has even become a virtue (Kakutani, 2017). Clearly what is needed is a cultural shift to where science, data, and the reliability of evidence are always included in discussions so that they can play a central role in working towards a sustainable and more socially just future. The coronavirus pandemic has perhaps begun to provide this shift, given the trust shown in the health advice for managing its spread, with hopes pinned on the effectiveness and equitable distribution of vaccines (World Health Organization, 2020).

## Statistical literacy, data literacy, and the practice of statistics

Statistics education, as used in the title of this paper, encompasses a range of definitions, interpretations, and suggestions for implementation at the school level. The background can be traced to Wild and Pfannkuch (1999), who described the work of their applied statistician colleagues with the PPDAC model: problem, plan, data, analysis, conclusion. The amplification of the parts of the model depends on the theoretical background and technical skills available to the user at the different stages of the model.

Research on the outcomes for school students related to the teaching and learning of statistics at school has progressed far in recent years, thanks to the recognition that it is not necessary to wait for theoretical statistics, *p*-values, and confidence intervals, to employ the PPDAC model and make decisions about questions involving data. Makar and Rubin (2009) led the way in suggesting *informal* inference, where a decision is made based on a question about a population using evidence from a sample, acknowledging uncertainty. This foundation was extended by Makar et al. (2011) and Ben-Zvi et al. (2012). The tools to use in gaining evidence become more complex over the school years. Watson et al. (2018) provided a detailed background from research on this process as the Practice of Statistics, whereas Franklin et al. (2007) created the *Guidelines for Assessment and Instruction in Statistics Education (GAISE) Report,* defining the Practice as follows:

1. Formulating questions, anticipating variability (P)
2. Designing and implementing a plan to collect data, acknowledging variability (P,D)
3. Analysing data with appropriate graphical and numerical methods, accounting for variability (A)
4. Interpreting the results of the analysis in relation to the original question, allowing for variability (C)

The framework, now updated to GAISE II by Bargagliotti et al. (2020), not only provides a structure for working with statistics but also incorporates the very foundation of statistics: variation (Cobb & Moore, 1997).

Attaching the word “literacy” to “statistics” and “data” has taken on different connotations for the two new phrases. Watson (2006) suggested that statistical literacy, although built on the Practice of Statistics through experiences at school, was the resultant critical thinking needed for citizens to make judgments when presented with the results and claims from the investigations of others:

Statistical literacy is the meeting point of the data and chance curriculum and the everyday world, where encounters involve unrehearsed contexts and spontaneous decision-making based on the ability to apply statistical tools, general contextual knowledge, and critical literacy skills. (p.11)

Based on examples from the media, Watson (1997) outlined the levels of engagement associated with statistical literacy. Tier 1 is the basic understanding of probabilistic and statistical terminology to apply the tools. Tier 2 is an understanding of probabilistic and statistical language concepts when they are embedded in the context of wider social discussion. Tier 3 is a questioning attitude that can apply more sophisticated concepts to contradict claims made without proper statistical foundation (p. 108).

Wolff et al. (2016), in reviewing the status of a definition of data literacy, employed the PPDAC model to characterise the many and varied suggestions in the literature, finding that all five steps of the model were relevant across the definitions. For Wolff et al., data literacy then made different contributions for four categories of citizens: Communicators, Readers, Makers, and Scientists. After also reviewing various teaching approaches, they proposed the following definition:

Data literacy is the ability to ask and answer real-world questions from large and small data sets through an inquiry process, with consideration of ethical use of data. It is based on core practical and creative skills, with the ability to extend knowledge of specialist data handling skills according to goals. These include the abilities to select, clean, analyse, visualise, critique and interpret data, as well as to communicate stories from data and to use data as part of a design process. (p. 23)

The Watson definition of statistical literacy is intended to apply to students as they leave school, most closely associated with Wolff et al.’s category of Readers: those “who need skills to interpret data that is [sic] increasingly presented as part of their every day life” (p. 18). Accordingly, mirroring in many ways the approach in the *GAISE Reports* (Bargagliotti et al., 2020; Franklin et al., 2007), it appears that data literacy is a preparation for statistical literacy.

## Where should statistics fit in the school curriculum?

**Fig. 1:**
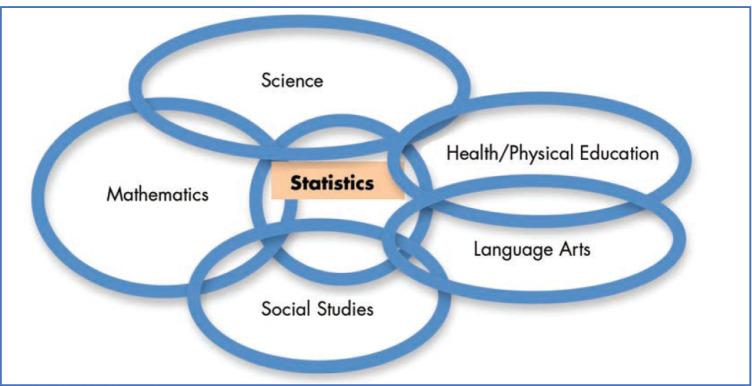
Teaching statistics across the curriculum (Usiskin, 2015, p. 12)

Although usually considered as a sub-strand of the discipline of mathematics in curriculum documents (e.g. Australian Curriculum, Assessment & Reporting Authority [ACARA], 2022), there are those who argue strongly that statistics is a separate discipline (e.g. MacGillivray, 2019; Royal Statistical Society, 2018), and in New Zealand, statistics has achieved equal status with mathematics in the school curriculum (Ministry of Education, 2017). Often, the difference between mathematics and statistics is associated with the contrast of “certainty”, arising from the concept of proof in mathematics, and “uncertainty”, arising from the concept of inference and its related “level of confidence” in statistics. This is an important distinction and although statistics employs many mathematical tools and results, its ultimate aim is to make inferences for populations (i.e. contexts) where not all data are available and a level of uncertainty must be acknowledged in any decision made. The existence of subjects such as Advanced Placement Statistics (College Board, 2020) in the USA for the senior year of high school could be seen as supporting this discipline view. Usiskin (2015) considered this option of an “independent subject” as one of four possibilities for teaching statistics at the school level as part of an extensive account of how to deal with statistics in relation to the mathematics curriculum in the K-12 years of schooling. His other three options were to teach statistics within mathematics, to teach statistics as applied mathematics, and to teach statistics across the curriculum as illustrated in Fig. 1. This cross-curriculum view would appear to align well with the early views of Freudenthal (1968, 1979) related to Realistic Mathematics Education, as well as with the more critical views of Gutstein (2006), Frankenstein (2012), D’Ambrosio (1990), and Skovsmose (1994) in terms of mathematics and issues that can be raised across the entire curriculum. The suggestion, which we strongly support, provides a transition to considering the current presence of reference to data across national curricula and examples from the classroom, as we expand below.

## The current status of statistics and in particular data across the curriculum

In a similar manner as Usiskin sees statistics across the curriculum, the *Australian Curriculum* (ACARA, 2022) views Numeracy, more generally, as one of the seven General Capabilities, essential across the curriculum: “fundamental to a student’s ability to learn at school and to engage productively in society”. Within the description of Numeracy, there are subsections for “Understanding chance” and “Interpreting and representing data”, which are described across the year levels, Foundation to 10. Although these descriptions include suggestions for actually dealing with data over the years, they also include the critical thinking of statistical literacy by including the evaluation of claims based on data in media reports, the recognition of bias in survey data, and the identification of sources of uncertainty in scientific investigations. The necessity for such a General Capability related to data is recognised across other parts of the school curriculum. A search of the *Australian Curriculum* reveals “data” mentioned 15 times in Foundation (F) to Year 10 Science, 26 times in F-10 Digital Technologies, 14 times in F-Years 6/7 Humanities and Social Science, and 39 times in Years 7–10 Geography (ACARA, 2019). It is a direct recognition of the need for the Practice of Statistics (Watson et al., 2018), the GAISE framework (Bargagliotti et al., 2020; Franklin et al., 2007), and statistical and data literacy (Watson, 1997; Wolff et al., 2016).

In the USA, the Standards for Mathematical Practices, within the *Common Core State Standards for Mathematics* (Common Core State Standards Initiative, 2010), present a similar stance to the Numeracy General Capability in Australia, for example, including practices related to graphing data in order to “search for regularity or trends”, to compare predictions with data, and to reason “inductively about data, making arguments that take into account the context from which the data arose” (p.7). A search of the US National Research Council’s (NRC) *Framework for K (Kindergarten)-12 Science Education* (2012) reveals 103 references to “data” across nine of the document’s 13 chapters. Furthermore, the *Next Generation Science Standards* (NGSS) (NRC, 2013) cover not only Science but also Engineering and Technology (p. 1), with emphases throughout on “analysing and interpreting data” as a Scientific and Engineering Practice (p. 3), and reference to data appearing across all topics and grade levels. As well as under the specific heading related to data, the “planning and carrying out investigations” heading often includes reference to planning investigations, e.g. “based on fair tests, which provide data to support explanations or design solutions” (p. 5).

In presenting its recent challenge to bring about change in high school mathematics learning in the USA, the National Council of Teachers of Mathematics (2018, pp. 57–64) included three foci related to essential concepts in statistics: Quantitative Literacy, reflecting the concerns of Frankenstein (2012), Gutstein (2006), and others on the importance of context; Visualising and Summarising Data, focussing directly on data in context; and Statistical Inference, creating a larger foundation for the application of the Practice of Statistics (Bargagliotti et al., 2020; Watson et al., 2018). Although still within Mathematics, there appears to be a recognition of context across the curriculum. Along with the data “on data” in other subject areas, perhaps the future will see more purposeful integration in schools. Beyond the school curriculum and classroom, challenges also exist for universities in preparing teachers for these expectations, as well as for educating graduates across many fields to address future pandemics and climate change (Lee & Power, 2021; Power, 2019).

## Data in context in the classroom

The previous section on curricula provides numerous hints as to where other subject areas provide topics that can be employed and linked to data handling and informal inference at the school level. In this section, we provide some explicit examples of classroom activities in meaningful contexts linking to data from across the curriculum relating to our concerns about the crises facing the world. These examples illustrate not only the posing of questions requiring data but also the need for accurate and reliable samples, for awareness of differing contexts when making comparisons, and for acknowledging possible variability in the conclusion reached.

Beginning with global climate issues, the S*cience Teacher*, for example, links its suggested topics directly to the NGSS (NRC, 2013). Hoover (2019) suggests a teacher-led, collaborative, inquiry-based series of lessons for high school students working in groups, to develop models of the carbon cycle, to refine them after peer feedback, and then to consider the data required to lobby their local community about the dangers of climate change, and encourage citizens to act to decrease the town’s greenhouse gas output. In a similar vein, climate change is the focus of a project-based learning sequence with a high school class working in three groups on different aspects of climate change using updated, comprehensive data for reports (Colaianne, 2015). Another angle on climate change is suggested by Krim and Brody (2008) in modelling the use of ice core samples to judge the presence of volcanic activity in past times, with students collecting data from simulated cores in the classroom. Pollution is the social and environmental issue addressed by Loux and Gibson (2019), who use an incident of lead poisoning of a city’s water supply to suggest an introductory activity with descriptive statistics revealing questions of sampling and ethical decisions in reporting results.

Looking more widely at climate change and sustainability from a geography perspective, Jones and Bagheri (2019) suggest a three-lesson unit for high school exploring national warming in 18 regions of the world, including South Central Asia, Southeast Asia, East Asia, and Oceania, in relation to climate change. Differences across the world are considered in relation to percentage change in carbon dioxide and methane for the regions, including maps by country, and figures highlighting the impact of oil extraction and refining, agricultural production, population, fossil fuel energy consumption, and renewable energy.

More specifically, Hendrickson (2015) describes an activity for middle school students related to their community’s involvement with fracking, a controversial hydraulic method of extracting gas from shale, and Countryman (2019) introduces proxy data, in the form of tree rings, to study climate change with high school students. In Australia, the *Australian Curriculum* (ACARA, 2019) has three Cross-curriculum Priorities, one of which is Sustainability. One of the Learning Area Statements across all learning areas in relation to the priority is:

Mathematical understandings and skills are necessary to measure, monitor and quantify change in social, economic and ecological systems over time and statistical analysis enables the prediction of probable futures based on findings and helps inform decision-making and actions that will lead to preferred futures.

With this priority in mind, Watson and English (2015) described working with Year 5 students asking first if their classes were environmentally friendly, and then extending the question to all Year 5 students in Australia using samples from an Australian Bureau of Statistics student “population”. The breath of topics linking opportunities for statistical investigations and statistical literacy to the Cross-curriculum Priority of Sustainability can also be employed for topics related to the other two Priorities: Aboriginal and Torres Strait Islander Histories, and Cultures and Asia and Australia’s Engagement with Asia (ACARA,^ 2022^).

Turning to topics related to COVID-19 itself, Stor and Briggs (1998) suggest an activity for high school students using dice to model the spread of a disease, using AIDS and the common cold as examples. More extensive suggestions for units for high school students are suggested by McClamroch and Montgomery (2009), who employ an on-line game called “Outbreak at Watersedge” to model disease spread, as part of an extensive 6-week summer programme for high school students, including each choosing an infectious disease to study. D’Agostino (2018) reports on a framework with aspects of connecting epidemiology to the real world, correlation not being equal to causation, making evidence-based arguments, designing a population-based study, and gathering data in the community (case–control studies), based on work with high school students.

More broadly across the curriculum, data are present in many contexts also related to issues potentially challenging the world students will enter after school. Issues associated with what data to collect are also significant in developing countries, such as Bangladesh. In the G*eography Teacher*, Lee-Ammons and Riosmena (2019) describe the issues associated with collecting census data in a very densely populated country challenged by a low-lying and constantly changing landscape (yet again suggesting links to climate change). *Teaching Statistics* also focuses on cross-curriculum topics in the social sciences, encouraging mathematics/statistics teachers to focus on meaningful contexts. For example, Brown (2017) uses student responses to national surveys on social issues, e.g. “why do you think there are people who live in need?”, to ask students to analyse and compare their class results with those in the public more generally. An angle on History is to use evidence, often involving data, to make a decision about which of two accounts of an event is most feasible. Leff (2019) describes presenting students with two scenarios for what happened over the centuries on Easter Island and to its inhabitants. As is often the case, the explanation of historical data may be contradictory when interpreted in more than one feasible context. In terms of teacher education in the Practice of Statistics, Hestness et al. (2014) provide the basis for a professional development programme for science teachers on climate change, again imbedded in the NGSS.

## Concluding remarks

The title of this paper purposefully refers to disruption and “crises”. Those who believe that once the COVID-19 crisis is overcome with a vaccine made available world-wide, life will return to the previous economic model of growth and prosperity are more than likely to be disappointed. The data from around the world, as well as within segments of the most prosperous societies, indicate that social disadvantage as well as the continued exploitation of earth’s unsustainable resources are going to continue to require global action (Davis, 2020; Mair, 2020; United Nations, 2015). The connected hyperobjects of the coronavirus pandemic and climate change have certainly presented the world with “real problems” involving high levels of uncertainty and enabled a once in a lifetime opportunity to promote the need for a statistically literate populace, with far reaching implications for the mathematics K-12 curriculum and beyond. Concerns still exist, for example in Australia, because national testing in mathematics only tests a few isolated data skills, upon which students do relatively well; hence statistics receives very little attention in the classroom, often relegated to Friday afternoon in primary school or the end of the year in high school (Watson & Callingham, 2020).

The arguments and evidence presented here should provide motivation for governments to develop educational policies in all countries to increase the focus on statistics, not only as part of mathematics but also across the curriculum. The Practice of Statistics should indeed be part of the recommended practice in school education wherever data-based questions are relevant in context and informal inferences can be made.

## Data Availability

Not applicable.
